# Associations between peak expiratory flow and frailty in olderly individuals: findings from the China health and retirement longitudinal study

**DOI:** 10.3389/fpubh.2024.1392581

**Published:** 2024-05-28

**Authors:** Rui Wang, Wen Shi, Wenlu Zhou, Yawen Xu, Junjie Wang

**Affiliations:** School of Nursing, Zhejiang Chinese Medical University, Hangzhou, China

**Keywords:** respiratory function, peak expiratory flow, frailty, older adults, CHARLS

## Abstract

**Purpose:**

Peak Expiratory Flow (PEF) is associated with a variety of adverse health outcomes in older adults; however, the relationship between PEF and frailty remains uncertain, and this study investigated the relationship between PEF and frailty within an olderly Asian demographic.

**Methods:**

Data were sourced from the Chinese Health and Retirement Longitudinal Study (CHARLS). Individuals in the study, all 60 years or older, underwent baseline PEF assessments quantified as standardized residual (SR) percentile values. The evaluation of frailty was conducted based on the criteria established by Fried. Participants without frailty at the outset were tracked over a four-year period, during which the relationships between PEF and frailty were examined through logistic regression and discrete-time Cox regression analyses.

**Results:**

Among 5,060 participants, cross-sectional analysis revealed that the prevalence of frailty was 2–3 times higher in the lower 10–49th and < 10th SR percentile groups compared to the 80–100th SR percentile group. The longitudinal study corroborated these results, showing an adjusted hazard ratio (HR) of 2.01 (95% CI, 1.15–3.51) for PEF SR percentiles below the 10th, in contrast to those between the 80th and 100th percentiles.

**Conclusion:**

PEF independently predicts and determines frailty in older adults. Declines in PEF greater than expected are associated with the development of frailty. Subsequent studies are encouraged to delve deeper into the connection between respiratory function and frailty in diverse contexts.

## 1 Introduction

Population aging is now a global phenomenon, with China experiencing the largest aging population worldwide. As per the 2020 national census data, China has over 260 million individuals aged 60 and above, presenting a significant challenge to the healthcare system (1). Among the various issues arising from population aging, the clinical condition of frailty stands out as particularly problematic (2, 3).

Frailty primarily denotes a non-specific state characterized by multiple functional abnormalities or diminished physiological reserves, leading to heightened vulnerability and reduced stress resilience in individuals (2, 4). This condition escalates dependence, fragility, and mortality risk (5). Compared to their non-frail counterparts, frail olderly individuals are more susceptible to adverse health outcomes, including falls, disability, delirium, emergency hospital admissions, and even death (6). Hence, early screening and diagnosis of frailty in olderly adults are crucial for averting these negative consequences. Age-related declines in lung function and respiratory strength, associated with health issues like diminished physical capacity and increased mortality risk (7, 8), may serve as indicators of frailty and its implications. However, the gold standard for lung function assessment, spirometry, necessitates specialized training and equipment (9, 10).

Peak expiratory flow (PEF), the maximum instantaneous flow achieved during forced expiration at maximal lung inflation, is notably simple and cost-effective (11, 12). Its measurements can be easily obtained even by untrained individuals, making it a practical tool for large-scale studies involving older adults. Prior research has identified a two-way relationship between frailty and respiratory illnesses, particularly those characterized by restrictive and obstructive patterns (13). However, there is limited evidence linking lung function, specifically PEF, with frailty in older adults (14, 15). Furthermore, while PEF has been associated with frailty in older adults in prospective cohorts, there is a notable gap in such data from Asian populations.

Our hypothesis posits that PEF values lower than anticipated could be linked to both the presence and progression of frailty in older adults. To explore this, our study utilizes data from the China Health and Retirement Longitudinal Study (CHARLS) to examine these hypotheses. This research marks the first instance of investigating the relationship between low PEF and the onset of frailty in community-dwelling older adults in China.

## 2 Methods

### 2.1 Study population

Our study’s data was sourced from the China Health and Retirement Longitudinal Study (CHARLS), a project aimed at collecting detailed and nationally representative data on households and individuals over the age of 45 in China, primarily to examine the dynamics of aging within the country. Detailed information about the CHARLS design and methods has been previously described in detail (16). In brief, CHARLS initiated a national baseline survey in 2011 (wave 1), followed by subsequent follow-up visits in 2013 (wave 2), 2015 (wave 3), and 2018 (wave 4). This baseline survey encompassed 450 villages and urban residences across 150 counties and districts, successfully interviewing 17,708 individuals who broadly represent China’s middle-aged and olderly adults. CHARLS received ethical approval from the Ethics Review Committee of Peking University (IRB0000105211015), and all participants provided written informed consent.

**Figure 1:**
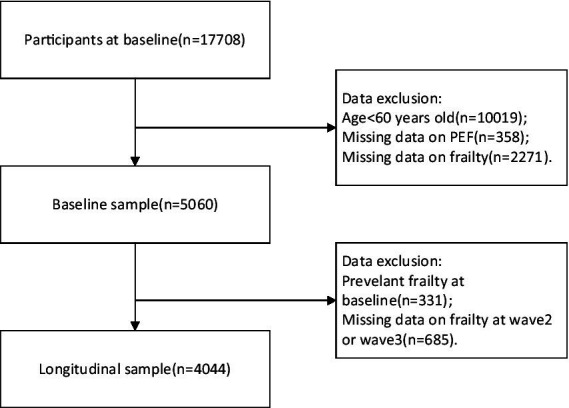
Flow diagram of participants selection.

In our study, we utilized data exclusively from wave 1, wave 2, and wave 3, as the collection of frailty information in wave 4 was incomplete. To specifically target the frail olderly demographic, we included only individuals aged 60 years and older. We excluded data from individuals who either lacked PEF information or whose frailty status could not be ascertained in the baseline analysis. Additionally, participants identified as frail at baseline were excluded from the analysis predicting the development of frailty, based on follow-up data from wave 2 and wave 3 (refer to Figure 1).

### 2.2 Measures

#### 2.2.1 Frailty

Frailty assessment in this study was conducted using the revised Physical Frailty Phenotype (PFP) scale (17, 18). This scale includes five criteria: weakness, slow walking speed, exhaustion, weight loss, and low physical activity (19, 20). Individuals meeting three or more of these criteria were classified as frail, while those meeting fewer were categorized as non-frail.

1. Weakness: This was assessed using the highest value from two grip strength tests performed with either hand while standing. Participants were classified as exhibiting weakness if their grip strength was at or beneath the 20th percentile, with adjustments made for gender and body mass index.
2. Slow Walking Speed: Defined as the average of two walking tests over a distance of 2.5 meters. Walking velocities that were at or under the 20th percentile, once adjusted for gender and height, were classified as reduced.
3. Exhaustion: Evaluated using two components from the Center for Epidemiologic Studies Depression Scale (CESD) (21). Participants were identified as meeting the exhaustion criterion if they indicated feeling “Occasionally or a moderate amount of time (3–4 days)” or “Most or all of the time (5–7 days)"when answering either of these questions: “I feel everything I did was an effort” or “I could not get going.”
4. Weight Loss: Participants self-reporting a loss of 5 kg or more over the past year during wave 1 and wave 2 were considered to have experienced significant weight loss. In wave 3, this criterion was met if there was a weight loss of 5 kg or more between wave 2 and wave 3.
5. Low Physical Activity: Defined based on participants’ responses to three questions. Participants were classified as having low physical activity if they answered “no” to all of the following questions: “During a usual week, did you do any vigorous activities for at least 10 min continuously,” “Did you do any moderate physical effort for at least 10 min continuously,” and “Did you do any walking for at least 10 min continuously.”

#### 2.2.2 PEF

PEF assessments were carried out with the use of a peak lung flow meter (EverpureTM, Shanghai, China). Participants were directed to stand, inhale deeply, securely enclose the mouthpiece with their mouths, and then exhale forcefully and rapidly. This procedure was repeated thrice, with 30-s intervals between each attempt. The highest reading from these three attempts, expressed in liters per minute, was used for analysis (22). Previous studies indicate that PEF naturally declines with age and varies among individuals due to factors like gender and height (11). Consequently, a reference group was formed by choosing a healthy subset from the same demographic, comprising individuals who had never smoked and had no history of diagnosed respiratory disorders, cardiovascular diseases, cancer, and similar conditions. This allowed us to use age, gender, and height-standardized PEF values to estimate the expected PEF values. PEF was defined in terms of:

1. PEF Residuals: Defined as the variance between the observed PEF and the anticipated PEF, these residuals were classified into groups with 10 L/min decrement intervals.
2. Percent Predicted: This metric was derived from the ratio of the actual measured PEF to the expected PEF. Predicted percentages were categorized at 10% intervals of decrease, and grouped into thresholds of 50, 80, and 100%.
3. PEF Standardized Residual (SR) Percentiles: Calculated by standardizing the ratio (measured PEF – expected PEF)/standard deviation of the residuals. An SR of 0 was equated to the 50th percentile. Percentiles were regarded as continuous variables and were grouped into categories decreasing by 10 percentage points, further divided into the following ranges: below 10th, 10th to 49th, 50th to 79th, and 80th to 100th percentiles.

### 2.3 Covariates

The study’s covariates included both demographic and health and functioning variables. Demographic variables comprised age, gender, residence (urban or rural), education level (uneducated, did not complete elementary school, elementary school, middle school, high school, and above), and marital status (married, widowed, or other). Health and functioning variables encompassed smoking status (never smoked, former smoker, current smoker), alcohol consumption (never, occasional, or regular), number of chronic conditions, cognitive functioning, and depression (yes or no). Occasional drinking was characterized as consuming alcohol fewer than once per month over the previous year, while regular consumption meant drinking more than once a month. Chronic diseases were identified based on self-reported histories of conditions like hypertension, diabetes mellitus, malignant neoplasms, chronic lung disease, liver disease, heart disease, kidney disease, stomach or digestive disease, stroke, arthritis, or rheumatism. Participants were grouped based on the number of chronic conditions (0, 1, and > 1). Cognitive functioning was evaluated using memory and mental state scores (23, 24), where the total cognitive score, ranging from 0 to 21, is the sum of these scores, with higher scores indicating better cognitive performance. Depression was assessed using the CESD-10 scale (25), with a score of 12 or more indicating depression (26).

### 2.4 Data analyses

Descriptive analyses were presented as means ± standard deviation for continuous variables and as frequencies and percentages for categorical variables. The baseline characteristics related to frailty status and covariates were categorized based on PEF SR percentile intervals. Interindividual characteristics were compared using the Kruskal–Wallis test and chi-square tests, as appropriate. Logistic regression analysis was utilized to assess the correlation between PEF and initial frailty, whereas discrete-time Cox regression analysis was applied to determine the connection between PEF and frailty in subsequent follow-up data. In this analysis, participants who completed the follow-up without becoming frail served as the reference group. The multivariate analysis comprised three models: Model 1 was unadjusted; Model 2 adjusted for age and gender; and Model 3 further adjusted for place of residence, education level, marital status, smoking status, alcohol consumption, number of chronic diseases, cognitive functioning, and depression.

To investigate whether baseline chronic lung disease or smoking habits influenced the relationship between peak expiratory flow (PEF) and frailty, participants were stratified into two subgroups in the fully adjusted model for a sensitivity analysis. Based on this stratification, interactions were assessed by integrating PEF and its corresponding multiplicative interaction factors into a comprehensively adjusted Cox regression model.

Each statistical test was two-sided, with a *p*-value of less than 0.05 indicating statistical significance. All statistical analysis was performed retrospectively with Stata 18 (Stata Corp, College Station, TX).

## 3 Results

### 3.1 Baseline sample characteristics

Table 1 displays the initial characteristics of the study cohort. Following the preliminary screening, a total of 5,060 individuals were enrolled in the study. Of these, 1,055 (20.8%) were classified in the 100th–80th PEF SR percentile, 1,544 (30.5%) in the 79th-50th percentile, 1970 (38.9%) in the 49th-10th percentile, and 491 (9.7%) in the <10th percentile. The average age of the participants was 67.6 ± 6.4 years, with 49.2% being female. Participants in the lower PEF SR percentiles, compared to those in the highest percentile group, were more likely to live in rural areas and have current smoking habits and regular alcohol consumption. Regarding health status, lower PEF SR percentiles were associated with a greater number of chronic diseases and a higher prevalence of depression. A total of 331 individuals were identified with debilitation at baseline, with the prevalence of debilitation increasing progressively with decreasing PEF SR percentiles (2.8, 5.9, 8.4, and 9.0%, respectively, *p* < 0.001).

**Table 1:** Baseline characteristics of study sample grouped by peak expiratory flow (PEF) standardized residual percentiles (*N* = 5,060).

Characteristics		All	Peak expiratory flow SR percentiles				*p*-value
		(*n* = 5,060)	80th–100th (*n* = 1,055)	50th–79th (*n* = 1,544)	10th–49th (*n* = 1970)	<10th (*n* = 491)	
Age, mean ± SD		67.6 ± 6.4	67.9 ± 6.6	67.5 ± 6.4	67.9 ± 6.4	65.9 ± 5.1	<0.001
Gender, *n* (%)	Male	2,572 (50.8)	580 (55.0)	664 (43.0)	940 (47.7)	388 (79.0)	<0.001
	Female	2,488 (49.2)	475 (45.0)	880 (57.0)	1,03 0(52.3)	103 (21.0)	
marital status, *n* (%)	Married	4,061 (80.3)	874 (82.8)	1,192 (77.2)	1,579 (80.2)	416 (84.7)	<0.001
	Widowed	925 (18.3)	168 (15.9)	330 (21.4)	367 (18.6)	60 (12.2)	
	Others	74 (1.5)	13 (1.2)	22 (1.4)	24 (1.2)	15 (3.1)	
Residential area, *n* (%)	Rural	3,177 (62.8)	613 (58.1)	982 (63.6)	1,275 (64.7)	307 (62.5)	0.004
	Urban	1883 (37.2)	442 (41.9)	562 (36.4)	695 (35.3)	184 (37.5)	
Education, *n* (%)	No formal education illiterate	1784 (35.3)	284 (28.3)	585 (39.4)	776 (37.9)	139 (26.9)	<0.001
	Did not finish elementary school	1,058 (20.9)	101 (20.6)	436 (22.1)	322 (20.9)	199 (18.9)	
	Elementary school	1,270 (25.1)	149 (30.4)	471 (23.9)	358 (23.2)	292 (27.7)	
	Middle school	637 (12.6)	70 (14.3)	209 (10.6)	192 (12.4)	166 (15.7)	
	High school or above	311 (6.2)	32 (6.5)	78 (4.0)	87 (5.6)	114 (10.8)	
Smoking, *n* (%)	Never smoked	2,890 (57.1)	610 (57.8)	967 (62.6)	1,129 (57.3)	184 (37.5)	<0.001
	Have quit	597 (11.8)	149 (14.1)	139 (9.0)	214 (10.9)	95 (19.4)	
	Current smoker	1,573 (31.1)	296(28.1)	438(28.4)	627(31.8)	212(43.2)	
Drinking, *n* (%)	Never	3,457 (68.3)	689 (65.3)	1,114 (72.2)	1,363 (69.2)	291 (59.3)	<0.001
	Occasionally	357 (7.1)	104(9.9)	95(6.2)	123(6.2)	35(7.1)	
	Regularly	1,246 (24.6)	262 (24.8)	335 (21.7)	484 (24.6)	165 (33.6)	
Number of chronic diseases, *n* (%)	0	1,363 (26.9)	312 (29.6)	423 (27.4)	513 (26.0)	115 (23.4)	0.002
	1	1,534 (30.3)	335 (31.8)	490 (31.7)	573 (29.1)	136 (27.7)	
	>1	2,163 (42.8)	408 (38.7)	631 (40.9)	884 (44.9)	240 (48.9)	
Cognition score, median (IQR)		9.5 (6.0, 13.0)	11.0 (8.0, 14.5)	9.5 (6.0, 13.0)	8.5 (5.0, 12.0)	10.0 (6.5, 13.0)	<0.001
Depression, *n* (%)	No	3,614 (71.4)	822 (77.9)	1,134 (73.5)	1,306 (66.3)	352 (71.7)	<0.001
	Yes	1,446 (28.6)	233 (22.1)	410 (26.6)	664 (33.7)	139 (28.3)	
Frailty, *n* (%)	No-frail	4,729 (93.5)	1,025 (97.2)	1,453 (94.1)	1804 (91.6)	447 (91.0)	<0.001
	Frail	331 (6.5)	2.8	91 (5.9)	166 (8.4)	44 (9.0)	

SD, standard deviation; IQR, interquartile range.

### 3.2 Cross-sectional association between PEF and frailty

The fully adjusted cross-sectional regression analysis model illustrates this point: When PEF was analyzed as SR percentile (OR = 1.14, 95% CI: 1.09–1.19) or percent predicted (OR = 1.13, 95% CI: 1.08–1.18), the risk of frailty increased by approximately 14% for each 10-unit decrease in PEF. In comparison to the group with the highest PEF SR percentiles, individuals in the lower percentile groups (10th to 49th and below 10th SR percentiles) exhibited a 2–3 fold increase in the number of frail participants, as shown in Table 2, Model 3.

**Table 2:** Cross-sectional correlation between peak expiratory flow and frailty.

PEF measures	*n*	Odds ratios and 95% confidence intervals of frailty					
		Model 1	*p*-value	Model 2	*p*-value	Model 3	*p*-value
*PEF residual*							
Per each 10 L/min decrease	5,060	1.02 (1.01–1.02)	<0.001	1.02 (1.01–1.02)	<0.001	1.02 (1.01–1.02)	<0.001
*PEF SR percentile*							
Per each 10th decrease	5,060	1.14 (1.10–1.19)	<0.001	1.18 (1.13–1.23)	<0.001	1.14 (1.09–1.19)	<0.001
80th–100th	1,055	Reference		Reference		Reference	
50–79th	1,544	2.14 (1.24–3.04)	<0.001	2.21 (1.27–3.16)	<0.001	2.13 (1.18–3.07)	0.01
10th–49th	1970	3.14 (1.90–4.40)	<0.001	3.25 (1.94–4.55)	<0.001	2.52 (1.46–3.57)	<0.001
<10th	491	3.36 (1.76–4.97)	<0.001	4.86 (2.47–7.24)	<0.001	3.82 (1.85–5.80)	<0.001
*PEF percent predicted*							
Per each 10% decrease	5,060	1.16 (1.11–1.21)	<0.001	1.17 (1.13–1.22)	<0.001	1.13 (1.08–1.18)	<0.001
>100%	2,312	Reference		Reference		Reference	
80–100%	942	1.28 (0.85–1.71)	0.154	1.36 (0.89–1.83)	0.078	1.16 (0.75–1.58)	0.408
50–79%	1,223	1.81 (1.29–2.33)	<0.001	1.89 (1.34–2.43)	<0.001	1.54 (1.07–2.02)	0.006
<50%	583	3.10 (2.14–4.07)	<0.001	3.24 (2.21–4.28)	<0.001	2.39 (1.58–3.20)	<0.001

Model 1 is unadjusted. Model 2 is adjusted for age and sex. Model 3 is also adjusted for educational level, marital status, smoking habits, residential area, drinking, number of chronic diseases, cognition score and depression. PEF stands for peak expiratory flow; SR represents standardized residual.

### 3.3 Correlation between PEF and frailty in subsequent follow-up studies

Table 3 demonstrates the association between PEF and the development of frailty during the four-year follow-up period. Out of all participants, 236 (5.8%) developed frailty. For each 10-unit reduction in PEF SR percentile, there was a roughly 8% rise in the likelihood of developing frailty. Moreover, individuals in the 10th–49th and < 10th percentile groups had over twice the odds ratio (OR) of developing frailty compared to those in the highest PEF SR percentile group. When PEF was calculated as a percent of the predicted value, the 10th-49th and < 10th percentile groups showed about a 1.5-fold increase in frailty risk compared to the reference group.

**Table 3:** Longitudinal correlation between peak expiratory flow and frailty.

	*n*	Hazard ratios and 95% confidence intervals of frailty					
PEF measures		Model 1	*p*-value	Model 2	*p*-value	Model 3	*p*-value
*PEF residual*							
Per each 10 L/min decrease	4,044	1.01 (1.01–1.02)	<0.001	1.01 (1.01–1.02)	<0.001	1.01 (1.00–1.01)	0.002
*PEF SR percentile*							
Per each 10th decrease	4,044	1.09 (1.04–1.14)	<0.001	1.11 (1.06–1.16)	<0.001	1.08 (1.03–1.31)	0.002
80th–100th	895	Reference		Reference		Reference	
50–79th	1,251	1.74 (1.13–2.66)	0.011	1.75 (1.14–2.69)	0.01	1.62 (1.06–2.50)	0.028
10th–49th	1,514	2.29 (1.53–3.43)	<0.001	2.31 (1.54–3.45)	<0.001	1.89 (1.26–2.84)	0.002
<10th	384	1.91 (1.11–3.29)	0.019	2.41 (1.39–4.18)	0.002	2.01 (1.15–3.51)	0.014
*PEF percent predicted*							
Per each 10% decrease	4,044	1.10 (1.05–1.15)	<0.001	1.11 (1.06–1.17)	<0.001	1.07 (1.03–1.12)	0.003
>100%	1971	Reference		Reference		Reference	
80–100%	765	1.37 (0.96–1.96)	0.081	1.44 (1.01–2.05)	0.046	1.31 (0.92–1.87)	0.141
50–79%	935	1.72 (1.25–2.35)	0.001	1.75 (1.28–2.40)	0.001	1.49 (1.09–2.05)	0.014
<50%	427	1.83 (1.22–2.75)	0.003	1.91 (1.27–2.87)	0.002	1.53 (1.01–2.31)	0.044

Model 1 is unadjusted. Model 2 is adjusted for age and sex. Model 3 is also adjusted for educational level, marital status, smoking habits, residential area, drinking, number of chronic diseases, cognition score, and depression. PEF, peak expiratory flow; SR, standardized residual.

### 3.4 Sensitivity analysis

After stratifying participants based on the presence of chronic lung disease, a significant association was observed between peak expiratory flow (PEF) and frailty in participants without chronic lung disease, compared to those with the condition (*p* = 0.001; Table 4). Similarly, when analyzing the data stratified by smoking status, the results indicated comparable associations between PEF and frailties (Supplementary Table S1). Furthermore, the addition of a multiplicative interaction term between PEF SR percentile and chronic lung disease or smoking status to examine changes in the relationship between baseline PEF and follow-up frailty revealed no statistically significant interaction (*p* interaction >0.05).

**Table 4:** Stratification of participants based on the presence or absence of chronic lung disease and their associated peak expiratory flow and frailty.

PEF measures	No chronic lung disease	*p*-value	Chronic lung disease	*p*-value
	Odds ratios and 95% confidence intervals of frailty			
*Cross-sectional analysis*				
*n*	4,366		680	
*PEF residual*				
Per each 10 L/min decrease	1.016(1.010–1.022)	<0.001	1.011(0.997–1.025)	0.114
*PEF SR percentile*				
Per each 10th decrease	1.144(1.086–1.202)	<0.001	1.116(0.978–1.254)	0.081
*PEF percent predicted*				
Per each 10% decrease	1.138(1.083–1.193)	<0.001	1.101(0.977–1.225)	0.094
	Hazard ratios and 95% confidence intervals of frailty			
*Longitudinal analysis*				
*n*	3,541		503	
*PEF residual*				
Per each 10 L/min decrease	1.010 (1.004–1.016)	0.001	1.001 (0.984–1.018)	0.911
*PEF SR percentile*				
Per each 10th decrease	1.092 (1.037–1.149)	0.001	1.011 (0.868–1.177)	0.89
*PEF percent predicted*				
Per each 10% decrease	1.088 (1.035–1.144)	0.001	0.993 (0.864–1.142)	0.926

Model is adjusted for age, sex, educational level, marital status, smoking habits, residential area, drinking, number of chronic diseases, cognition score, and depression. PEF, peak expiratory flow; SR, standardized residual.

## 4 Discussion

Previous research has identified a link between frailty and PEF and has utilized PEF as a predictive indicator of frailty in older populations (11, 15, 27). Nonetheless, there is some controversy, as highlighted by the study of Charles et al., which did not find a correlation between PEF and frailty after adjusting for covariates (14). Moreover, most prior studies have been confined to cross-sectional designs and specific settings, such as nursing homes, with a notable lack of data from Asian populations, thus limiting the applicability of PEF in detecting frailty in these groups. This study, therefore, stands as the first to investigate the association between PEF and frailty using both cross-sectional and longitudinal approaches within a generalized Asian population, utilizing CHARLS data. Our findings indicate that lower-than-expected PEF values are independently associated with both the development and presence of frailty in olderly adults.

Various mechanisms, such as sarcopenia – a widespread reduction in skeletal muscle mass and function associated with aging, help elucidate the link between PEF and frailty (28–30). This condition leads to weakened muscles throughout the body, including the respiratory muscles (31–33), and plays a crucial role in the frailty indicators of the FRAIL scale, such as low grip strength and slow walking speed (34). Additionally, sarcopenia-induced respiratory muscle weakness significantly impacts peak respiratory flow rates. Studies have shown a longitudinal link between decreased PEF and sarcopenia (35, 36). Moreover, low physical activity and impaired respiratory function due to sarcopenia exacerbate fatigue, further increasing frailty risk and perpetuating a vicious cycle. Inflammation is another key mechanism contributing to respiratory impairment and frailty. Chronic inflammation drives age-related declines in physical functioning, leading to reduced mobility, cognitive impairment, and the development of both physical and cognitive frailty (37, 38). This inflammation not only weakens muscle strength but also impairs lung function (39, 40), which in turn increases the risk of lung infections and frailty in individuals with lower PEF. Furthermore, decreased lung function is linked to impaired cognitive function and a heightened risk of dementia (41, 42). Indications are that ongoing deterioration in lung capacity could lead to a state of inflammation driven by reduced oxygen supply to the brain (43), making individuals with low PEF more prone to developing frailty.

While the mechanisms discussed above indicate a bidirectional relationship between frailty and respiratory function, our longitudinal analysis excluded individuals already frail at baseline. Our results indicate that reduced PEF could act as an early indicator and contributing factor to frailty. The results of this study demonstrate that the impact of PEF on frailty in olderly adults remains consistent, irrespective of whether it is assessed at a single time point or observed over a period. This consistency suggests that the mechanisms linking these two variables are stable, and these determinants do not undergo significant changes over time. Previous research often indicates that certain relationships are enduring (12, 35). Our findings align with these studies and exhibit similar patterns in both cross-sectional and longitudinal analyses. Prior studies have indicated an increased association between frailty and PEF in the presence of chronic lung disease (7, 44). However, in this study, the fully adjusted model and subsequent sensitivity analysis revealed no significant findings. Furthermore, upon including interaction terms, no significant interactions between PEF and chronic lung disease were observed. This could be due to other covariates in our study that are closely linked with chronic lung disease, obscuring potential interactions. Additionally, despite the known impact of smoking on respiratory impairment, our study did not find any interaction between smoking status and PEF. This underscores the need for future research to further explore these associations in different contexts and populations, taking into account various symptoms related to respiratory disorders.

This study has certain limitations, including the exclusion of part of the sample due to missing information. This excluded subset may have been older and in poorer health, potentially leading to an underestimation of frailty prevalence. Additionally, while we controlled for numerous relevant covariates, unaccounted confounding factors might still exist, introducing potential bias. Furthermore, due to limitations inherent in the CHARLS database, our study exclusively employed the Revised Physical Frailty Phenotype (PFP) scale for frailty assessment and employed only PEF to evaluate respiratory function. This approach represents a potential bias. Future research could explore variations in outcomes derived from different frailty assessment methods, providing a detailed analysis of the relationship between individual frailty components and the Respiratory function assessment. However, the study also possesses notable strengths. As far as we are aware, this is the inaugural long-term study in Asia examining the link between PEF and the risk of frailty, utilizing a large, nationally representative sample from the CHARLS dataset, which includes community-dwelling older adults in China. Furthermore, the assessment of PEF through various methods enhances the reliability of our findings.

## 5 Conclusion

In conclusion, through both cross-sectional and longitudinal analyses, this study has established a clear relationship between decreased PEF and frailty in an Asian population. We have demonstrated that PEF can serve as an independent predictor of frailty among older adults, with lower-than-expected PEF values being notably associated with the development of frailty. Future research should focus on further exploring the impact of chronic respiratory diseases on the correlation between PEF and frailty.

## Data availability statement

The datasets presented in this study can be found in online repositories. The names of the repository/repositories and accession number(s) can be found below: https://charls.pku.edu.cn/.

## Ethics statement

The studies involving humans were approved by Peking University Biomedical Ethics Committee. The studies were conducted in accordance with the local legislation and institutional requirements. Written informed consent for participation was not required from the participants or the participants’ legal guardians/next of kin in accordance with the national legislation and institutional requirements.

## Author contributions

RW: Data curation, Writing – original draft, Writing – review & editing. WS: Software, Writing – review & editing. WZ: Methodology, Software, Writing – review & editing. YX: Software, Writing – review & editing. JW: Supervision, Writing – review & editing.
