# Supplementary material for: Associations between peak expiratory flow and frailty in olderly individuals: findings from the China health and retirement longitudinal study
Source: Front Public Health. 2024 May 28;12:1392581. doi: 10.3389/fpubh.2024.1392581 (PMC11165131; doi:10.3389/fpubh.2024.1392581)
Supplement: Supplementary file 1 [file Table_1.DOCX]

Supplementary Material

# Supplementary Figures and Tables

## Supplementary Tables

**Supplementary Table S1.**

Stratification of participants based on habits of smoking and their associated peak expiratory flow and frailty

|  | Odds Ratios and 95% Confidence Intervals of Frailty | | |  |
| --- | --- | --- | --- | --- |
| **PEF Measures** | never smoke | P-value | former or current smoker | P-value |
| **Cross-sectional analysis** |  |  |  |  |
| **n** | 2890 |  | 2170 |  |
| **PEF Residual** |  |  |  |  |
| Per each 10 L/min decrease | 1.017(1.010-1.025) | <0.001 | 1.011(1.003-1.019) | 0.007 |
| **PEF SR Percentile** |  |  |  |  |
| Per each 10th decrease | 1.161(1.091-1.232) | <0.001 | 1.103(1.027-1.180) | 0.005 |
| **PEF Percent Predicted** |  |  |  |  |
| Per each 10% decrease | 1.143(1.081-1.206) | <0.001 | 1.103(1.027-1.180) | 0.005 |
|  | Hazard Ratios and 95% Confidence Intervals of Frailty | | |  |
|  | never smoke | P-value | former or current smoker | P-value |
| **Longitudinal analysis** |  |  |  |  |
| **n** | 2279 |  | 1765 |  |
| **PEF Residual** |  |  |  |  |
| Per each 10 L/min decrease | 1.111(1.041-1.186) | 0.002 | 1.004(0.997-1.012) | 0.270 |
| **PEF SR Percentile** |  |  |  |  |
| Per each 10th decrease | 1.111(1.041-1.186) | 0.002 | 1.042(0.973-1.116) | 0.240 |
| **PEF Percent Predicted** |  |  |  |  |
| Per each 10% decrease | 1.096(1.032-1.164) | 0.003 | 1.039(0.968-1.115) | 0.293 |

Model is adjusted for age, sex, educational level, marital status, residential area, drinking, number of chronic diseases, cognition score and depression. Abbreviations: PEF, peak expiratory flow; SR, standardized residual.

**Supplementary Table S2.**

Multivariate regression models and predictive equations for peak expiratory flow from a subsample of healthy CHARLS participants

|  | Men(n=190) |  | Women(453) |  |
| --- | --- | --- | --- | --- |
|  | Coefficient(SE) | Mean(min-max) | Coefficient(SE) | Mean(min-max) |
| Intercept(L/min) | 134.68(227.527) | 307.45(30-690) | -164.69(107.88) | 218.61(30-560) |
| Age(years) | -6.99(1.22)*** | 69.28(60-89) | -2.61(0.55)*** | 68.52(60-95) |
| Height(m) | 404.59(118.35)** | 1.62(1.37-1.84) | 372.82(60.72)*** | 1.51(1.30-1.77) |
| Predictive equation | 134.68-6.99*Age+404.59*Height | | -164.69-2.61*Age+372.82*Height | |
|  | SDR=116.86 , R^2^=0.23 | | SDR=82.90 ,R^2^=0.15 | |

Abbreviations: SE, standard error; SDR, standard deviation of the residuals; R^2^, R-squared.

*p<0.05; **p<0.01; ***p<0.001.
